# 

**Published:** 1893-08-05

**Authors:** 


### CONTENTS.

**London Editors and London Hospitals** . . . . . 289

**Around the Hospitals** . . . . . . . . . . 290

**Diet in Disease.** By Mrs. Ernest Hart. XXVII.—Ulcer of the Stomach . . . . . . . . . . . 291

**Gleanings in Science and Medicine** . . . . . 292

**The Hospital Clinic—**

The Treatment of Appendicitis . . . . . . . . 297

Paddington Green Children's Hospital.—The Treatment of Pleurisy and Empyema (continued) . . . . . . 298

Antiseptic Treatment and Disinfection of Scarlet Fever: Remarks on the Method of Using Inunction . . . 299

**Famous Poisoners in Fiction.** I.—Introductory . . . 293

**What Becomes of Stillborn Children?** . . . . . 294

**Annotations.**—Health Resorts: A Protest—Cholera Precautions in Villages—Rich Deserters at West End Churches . . . 295

**Notes and News** . . . . . . . . . . . 296

**Metropolitan Hospital Sunday Fund** . . . . 300, 304

**The Institutional Workshop—**

The London Hospital and the "Pall Mall Gazette." By Our Special Commissioner . . . . . . . . 301

**Editor's Letter-Box.**—"Sanitas" Oil and Inunction . . 303

**The Book World of Medicine and Science** . . . vii

**Medical Vacancies, Appointments, &c.** . . . . ix

### EXTRA SUPPLEMENT.—THE NURSING MIRROR.

**News from the Nursing World.**—Incompetent to Criticise—Increased Accommodation for Nurses—Merits and Demerits—For Patients or Nurses?—No Fresh Air!—Whose Fault is it?—Valuable Lectures—Unbusiness-like Correspondents—Rural Nursing in Lincolnshire—Progress at Bangor—New York—An American Novelty—A New Home at Vancouver—English Nurses in Paris—Indian Nursing—Short Items—The Disabled Nurse . . . . . . clxxxi

**Sanitation for Nurses.** By A Sanitary Inspector.—XVI.—Methods of Disinfection in Foreign Countries . . . clxxxiii

**District Nursing.**—The Parish Nurse . . . . . clxxxiv

**Wants and Workers** . . . . . . . . . clxxxiv

**Our Examination Questions.**—Novelties for Nurses clxxxv

**Lady Nurses for Officers in India.**—By One Who Knows Them . . . . . . . . . . . . . clxxxv

**Queen's Nurses** . . . . . . . . . . . clxxxv

**Everybody's Opinion.**—The Cape Mission—Condy's Fluid—The London Hospital—Out-Door Uniform—Another Opinion clxxxvi

**Minor Appointments** . . . . . . . . . clxxxvii

**Free Lectures.** II.—Saline Solution . . . . . clxxxvii

**New York City Training School for Nurses** . . . clxxxvii

**For Reading to the Sick.**—Aspiration . . . . clxxxvii

**Appointments,** clxxxvii; **Notes and Queries** . . . clxxxviii

**The Muses' Looking-Glass.**—The Life and Work of John Ruskin . . . . . . . . . . . . . clxxxviii

**The Book World for Women and Nurses** . . . . clxxxix

### USED AND RECOMMENDED BY HIGH AUTHORITIES.

NONE GENUINE UNLESS BEARING THE TRADE MARK.

**SOLUTION D.**

For General Disinfecting Purposes.

**PERFECT DEODORIZER.**

**NON-POISONOUS.**

**NON-CORROSIVE.**

**DOES NOT STAIN.**

*SOLD EVERYWHERE.*

**1/- per Bottle.**

(*Champagne Quarts.*)

"AMINOL" — TRADE MARK — SOLE PROPRIETORS *The "Amines" Syndicate Lim.* 101 LEADENHALL STR. LONDON, E.C. — A TRUE DISINFECTANT

**SOLUTION R.**

For Use in Medicine and Surgery.

**AS DRESSING, MIXTURE, LOTION, GARGLE, SPRAY, OR INJECTION.**

An efficient Remedy in Suppurative, Phlegmonous, or Fermentative Disease-processes o the Skin, the Mucous Tract, etc.

*OF ALL CHEMISTS, etc.*

**Bottles, 1/1½, 1/9, 2/9.**

PROTECTED BY ROYAL LETTERS PATENT.

**SOLE AGENTS FOR UNITED KINGDOM:—**

### THOS. HODGKINSON, PRESTONS, & KING, 81, Bishopsgate St. Without, E.C.

SOLUTION "D."

The Surveyor to one of the largest London Vestries, writes 26 January, 1892:—"I have tested the qualities of the 'Aminol' Disinfectant, and find the results to be satisfactory. It is a thorough Disinfectant, *killing* and subduing all putrid and bad smells, and not merely hiding them, as is the case with many other disinfectants.

"I consider that, when its usefulness becomes known, it will be very much used."

SOLUTION "R."

This solution is in itself a perfectly harmless fluid as regards the human organism; therefore, when applied to wounds, no undesirable disturbances could ensue owing to its being absorbed; this is well known to be the case with some antiseptics—as in carbolic acid applications, or in the use of perchloride of mercury. Its function is that of a germicide, and, being applicable both externally and internally, as a Dressing, Lotion, Mixture, Gargle, Spray, or Injection, it is invaluable in all diseases caused by germs, *and the seat of which is accessible to the remedy.*

It has now been sufficiently tested by a large number of physicians and surgeons of standing (in various diseases of the skin and the mucous tract) to place its importance and its therapeutical value beyond doubt.

**Pamphlets, &c., on application.**

ORIGINALS OF ALL TESTIMONIALS CAN BE SEEN AT THE OFFICE OF

### THE "AMINES" SYNDICATE, LTD., 101, LEADENHALL STREET, E.C.

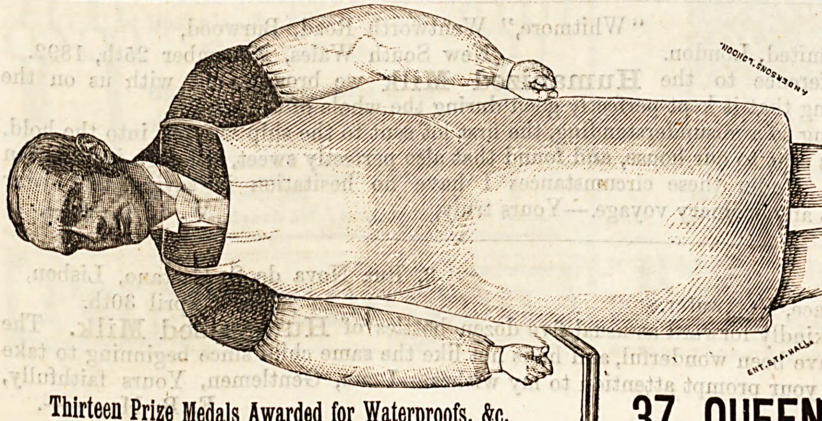

Thirteen Prize Medals Awarded for Waterproofs, &c.

### ANDERSONS' WATERPROOF APRONS AND SLEEVES FOR OPERATIONS.

The Waterproof Manufacturers,

### 37, QUEEN VICTORIA ST., E.C.

